# Venous Thromboembolism Prophylaxis in the Neurocritically Ill Population

**DOI:** 10.3390/jcm14134434

**Published:** 2025-06-22

**Authors:** Oyshik Banerjee, Roysten Rodrigues, Lauren Adkins, Katharina M. Busl

**Affiliations:** 1 Department of Pharmacy, Shands Hospital, University of Florida, Gainesville, FL 32610, USA; oyshik.banerjee@ufhealth.org; 2 Department of Neurology, Division of Neurocritical Care, College of Medicine, University of Florida, Gainesville, FL 32610, USA; 3 Librarian, Health Science Center Libraries, University of Florida, Gainesville, FL 32610, USA; lauren.adkins@ufl.edu; 4 Department of Neurosurgery, College of Medicine; University of Florida, Gainesville, FL 32610, USA

**Keywords:** VTE, intracranial hemorrhage, spinal cord injury, PTP, subdural hemorrhage, traumatic brain injury, subarachnoid hemorrhage, neurosurgery, neurocritical care, hematoma expansion, craniotomy

## Abstract

**Background/Objectives**: Venous thromboembolism (VTE) is a preventable cause of morbidity in the neurocritical ill patient population. There is ongoing debate regarding the optimal timing and choice of pharmacologic thromboprophylaxis (PTP) and how these decisions relate to balancing the risk of bleeding complications with the development of VTE. Our review assesses the available data to provide un updated perspective to clinicians. **Methods**: A literature search was performed in December 2024 in PubMed and EMBASE. We focused on the timing of PTP initiation and the comparison of enoxaparin (ENX) with unfractionated heparin (UFH) in patients with traumatic brain injury (TBI), intracerebral hemorrhage (ICH), subarachnoid hemorrhage (SAH), spinal or spinal cord injury (SCI), or requirement for neurosurgical intervention. **Results**: We included 90 articles spanning a total of 669,725 patients with injuries of interest within neurocritical care. The existing data largely signaled a benefit of early administration (<24–72 h) of PTP in VTE prevention, though some studies suggested increased risks of complications. Data to inform a preference for PTP agent was less robust, though a signal of benefit for enoxaparin is suggested for subsets of patients with acute brain injury such as TBI. The data quality is limited by the large body of retrospective studies, the heterogeneity of study populations, outcome definitions, study methodologies, and the lack of detailed reporting of relevant factors. **Conclusions**: Our review provides an updated assessment of the available data on PTP timing and choice in neurocritically ill patients with hemorrhages or surgical need, with a practice-focused overview for clinicians balancing VTE risk with bleeding risk. The data suggest that in most circumstances, early PTP appears safe and indicated, and that low-molecular weight heparin (LMWH) can be considered over UFH in certain subsets of patients. Still, data gaps and conflicting results highlight the need for patient-specific decision making and indicate that more robust research is warranted to inform optimal clinical practice.

## 1. Introduction

Balancing the prevention of venous thromboembolism (VTE) with pharmacologic thromboprophylaxis (PTP) and the risk of bleeding in patients with acute intracranial or intraspinal hemorrhages, or with increased risk of hemorrhage in the central nervous system, is a complex challenge. VTE is a leading cause of preventable death among critically ill patients [1]. Neurocritically ill patients carry an even higher VTE risk than the general critically ill population, with the prevalence ranging from 2.5% to 21%, even when including those with pharmacologic thromboprophylaxis (PTP) [2,3]. Omission of initiation of PTP in the first 24 h without documented contraindication is common and is associated with an increase in the odds of in-hospital-mortality [4]. VTE risk is often elevated in the context of fulfilling all three elements of Virchow’s Triad [5]: tissue injury and endothelial damage related to the primary injury or neurosurgical intervention (NI), hemostasis from immobility, and hypercoagulability [6].

While the VTE risk is considerably high, the implications of worsening hemorrhage can be dramatic as they risk worsened functional outcomes and mortality or may result in the need for repeat neurosurgical intervention. Characterizing optimal PTP practice in a neurocritical care population is complicated by the heterogeneity of these patients and their individual, diagnosis-specific bleeding and thrombotic risks. The Neurocritical Care Society and Society of Critical Care Medicine published recommendations on PTP in 2016 and 2017 [6,7], but there is a lack of more recent consensus VTE prophylaxis guidelines with definitive recommendations for neurocritical care patients. Due to a lack of data, existing guidelines vary in their guidance on the timing of PTP and the agent of choice (Table 1) [6,8,9,10,11,12]. This gap in the guidance likely contributes to the observed practice variability and uncertainty [2,13].

In this narrative review, we assess contemporary data with a focus on two pertinent questions regarding PTP in the neurocritical care population with high risk of hemorrhage (i.e., intracranial hemorrhage (ICH), traumatic brain injury (TBI), non-traumatic subarachnoid hemorrhage (SAH), spinal injury or spinal cord injury (SCI), and those who require neurosurgical intervention): is there a preferable time frame in which the evidence suggests to initiate PTP so as to minimize the risks of thrombosis and bleeding, and is there one agent that the evidence suggests as an optimal choice?

### Considerations for Choice of VTE Chemoprophylaxis Agent

The two most frequently discussed agents for PTP are unfractionated heparin (UFH) and enoxaparin (ENX), a low-molecular-weight heparin (LMWH) (Table 2). Hence, we focus on these two specific agents when considering the choice for PTP.

UFH has attractive characteristics for patients at risk of (worsening) bleeds: shorter half-life, which might be considered apt for patients who may need unexpected intracranial or spinal surgery, especially if related to acute bleeding; fully neutralizable with protamine; and safety with renal dysfunction. These properties of UFH must be weighed against the relative benefits of ENX. These include its lower risk of heparin-induced thrombocytopenia, consistently higher bioavailability, more predicable kinetics (owing to its consistently smaller size and lower protein binding), and its longer half-life, facilitating prolonged prophylactic effect [14,15,16].

## 2. Methods

The literature search of the bibliographic databases PubMed and EMBASE was performed by a health science librarian (L.E.A.) on December 13, 2024. The search strategy combined database-specific controlled vocabulary truncated and phrase-searched keywords in titles and abstracts, as available for the concepts venous thromboembolism, chemoprevention, prophylaxis, enoxaparin, unfractionated heparin, and neurocritical care patients. The search results were limited to the English language. We also manually searched relevant journals for articles to include in addition to results retrieved through searching the databases. The full search strategies are reported in the Supplementary Materials. We included studies comprising were prospective or retrospective primary analyses attempting to address the question of safety or efficacy of ENX or UFH with respect to timing or choice of agent. We excluded references that were published guidelines, meta-analyses or systematic reviews, and analyses that did not address the purpose of this review or were published only in abstract form or prior to 1998.

## 3. Results

**Figure 1:**
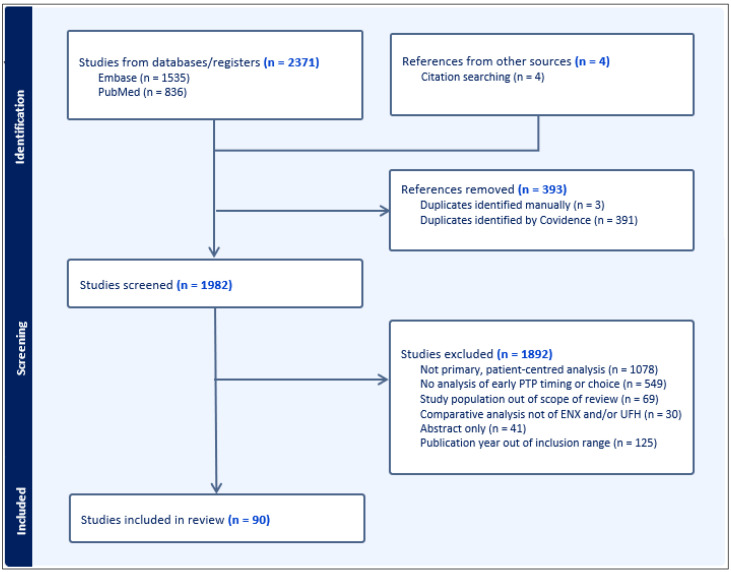
Prisma flow diagram of relevant studies.

The literature search yielded a combined 2371 records, of which 1982 remained after removing 393 duplicates (Figure 1). We included 90 articles for the review (Figure 1), totaling 669,725 patients included in relevant analyses with injuries of interest. Of these studies, 9 were prospective, randomized studies, and 8 were prospective, observational studies. The remaining 73 studies were retrospective, observational studies, of which 51 utilized either propensity score matching and/or multivariate regression analyses to account for presumed confounding variables to assess the independent association of an exposure and outcomes of interest. These statistical methods varied significantly, particularly in the chosen variables incorporated into multivariate analyses. Even after categorizing and reviewing these studies by included injury/diagnosis type, significant variance was evident in the timing of PTP initiation (as well as the definition of delayed and early PTP), choice or comparison of agents, dosing, and the severity of initial injury, among other baseline demographics, as well as the level of reporting of this information. These studies largely focused on the utilization of UFH and/or ENX, though some analyzed the use of LMWH without further characterization of the agent, or LMWH as mostly represented by ENX, with a minority utilization of alternative LMWH agents (i.e., dalteparin). The demographic data reported in this review reflects the matched populations where applicable, to best represent the populations for which matched cohort-adjusted results were presented in the literature. All the included studies are summarized in the supplementary excel file, with prospective studies separated from retrospective analyses to facilitate study navigation by design.

## 4. Discussion

### 4.1. Traumatic Brain Injury

TBI is a global public health problem. Men are affected more; peaks include children in the age group (0–4) and older adolescents (15–19). The growing aging population in the U.S. contributes to increasing frequency of TBI, with adults aged 75 or older requiring hospitalization more frequently [17].

The incidence of VTE in TBI patients has been reported to exceed 50% [18,19]. Risk factors in patients with isolated penetrating head injury include age, male sex, obesity, and hypertension. Those that require ICP monitoring and/or further NI (craniotomy) delay in initiation of PTP > 72 h [18,19]. Penetrating injuries to the abdomen, spine, or upper/lower extremity (1), head Abbreviated Injury Scale (AIS) > 3, shock on admission, mechanical ventilation > 3 days [20], >4 missed dose of PTP, increased length of hospital stay, history of IVC filter, and low Glasgow Coma Scale (GCS) (<8) on admission [21] have also been considered independent risk factors.

#### 4.1.1. Timing of PTP and Outcomes

Thirteen studies suggested benefit with “earlier” initiation associated with decreased risk of VTE without a signal for harm [18,20,22,23,24,25,26,27,28,29,30,31,32]. Six studies with PTP started < 72 h after injury [18,25,26,28,29,30], four < 48 h [20,22,24,27], two < 24 h [31,32], and one suggesting benefit with each hour PTP was expedited [23]. Fourteen studies did not detect benefit nor increased risk of bleeding complications [33,34,35,36,37,38,39,40,41,42,43,44,45,46], of which seven analyzed PTP within 24 h of complications [33,34,35,36,37,38,43], three within 48 h [39,42,44], and four within 72 h [40,41,45,46]. Ten studies incorporated repeat CT head to inform PTP [33,34,36,37,38,41,42,44,45,46]. Four studies suggested an association of PTP use with increased risk of bleeding complications [47,48,49,50]. One retrospective, propensity-matched analysis of 12,879 trauma patients with severe TBI found that PTP within 24 h was independently associated with a lower risk for VTE (2% vs. 3.5%; OR 1.76, *p* < 0.01) in all TBI patients (though not in isolated TBI), along with increased risk of requiring a non-fatal intracranial operation (all TBI patients: 1.8% vs. 0.41%, OR 0.22, *p* < 0.001; isolated TBI: 2.3% vs. 0.43%, OR 0.18, *p* < 0.001) [47]. Two single-center cohort studies found that in patients for whom initial repeat computed tomography (CT) showed ICH progression, subsequent early PTP significantly increased the risk of further worsening ICH on repeat CT, but with no such risk found in those whose initial follow-up CT was deemed stable [48,50].

#### 4.1.2. Choice of Agent for PTP and Outcomes

Twelve observational studies [18,20,22,23,24,29,32,39,47,49,50,51,52,53] suggested benefit with the use of LMWH (seven analyzing PTP < 24–48 h after injury and one reporting on early repeat CT utilization) related to an associated decrease in the risk of VTE [18,20,22,23,29,39,47,50,53] and/or mortality [18,23,24,29,32,49,51,53], with none suggesting an increased risk of bleeding complications. Eight retrospective studies found mortality benefit with LMWH, though two multicenter, matched cohort studies, one with 14,926 patients > 65 years old with isolated severe TBI (OR 0.81, *p* = 0.023) and another with 3320 patients with severe TBI and SDH (OR 0.480, *p* = 0.008), did not find an associated VTE risk reduction [24,51].

#### 4.1.3. Interpretation and Considerations

The interpretation of this data is challenged by the heterogeneity in the definition of early PTP, the reporting of bleeding complications (e.g., expansion of TBI related ICH reported in only 18 studies) and details characterizing the need for neurosurgical intervention, and the difficulty with generalizing findings due to variable and incompletely described population characteristics. Most studies are limited by retrospective, and many by single-center, design.

Taken together, PTP initiation < 48–72 h after TBI, contextualized by TBI severity and risk factors for higher VTE risk in this population, suggests VTE risk reduction. No consistent signal for a higher risk of harm was seen with PTP within 24 h in patients whose repeat CT suggested the stability of TBI-related bleeding, and thus it can be considered in this scenario, as guidelines have suggested [8,9], that LMWH use can be considered after analysis of patient-specific risks and benefits.

### 4.2. Intracranial Hemorrhage

ICH accounts for 10–30% of all strokes [54] and is associated with mortality rates of 30–40% [55]. Incidences of VTE, symptomatic DVT, and PE in hospitalized patients is about 2–4%, 1–2%, and 0.7–2%, respectively [54], with most occurring within the first 7 days of hospitalization. Identified risk factors for VTE include age group above 50, male sex, heart failure, atrial fibrillation, disorders of consciousness, larger hematoma volume (>15 mL), and length of hospital stay [56].

#### 4.2.1. Timing of PTP and Outcomes

Eight studies focused mostly on the safety of early PTP [57,58,59,60,61,62,63,64,65], including five which reported the utilization of early repeat CT to inform PTP, with none finding an associated increased risk of bleeding complications (namely hematoma expansion [HE]) [58,62,63,64,65]. Of note, two single-center studies evaluating the ultra-early initiation of PTP found no higher risk of hematoma expansion [63,66]. One study found PTP to be associated with increased risk of worse functional outcomes at three months [59]. A post hoc analysis of the INTERACT 2 study, a large prospective, multicenter, cohort study of 754 propensity score-matched patients enrolled <6 h after ICH, found PTP < 7 d after ICH to be associated with increased risk of major disability (mRS 3–5) at 90 d(OR 1;68, *p* < 0.001) vs. no PTP, as seen particularly in a subgroup analysis of patients with NIHSS < 15 [59]. PTP was also associated with a decreased risk of death in the overall population (OR 0.55, *p* = 0.01) (but not in survivors at 48 h), and the authors noted that PTP was not associated with greater rebleeding or HE [59]. Two meta-analyses on the timing of PTP after ICH evaluating a wide range of PTP initiation between 24 h and 7 d of injury found no major differences in risk of HE, death, or functional outcomes [67,68].

#### 4.2.2. Interpretation and Considerations

The interpretation of this data is complicated by the heterogeneity in the definition of early PTP, inconsistency in the reporting of important baseline characteristics (e.g., ICH score and GCS reported in only three and six studies, respectively), the definition of HE (some defined by any growth and others by different levels of expansion), and the description of a standard approach to repeat imaging. The generalizability of these studies is narrowed by the populations being characterized largely by supratentorial ICH with initial volumes < 25 mL. Most studies are limited by their single-center design and some by their retrospective design. Still, these data suggest PTP < 24–48 h after ICH (particularly with similar initial ICH volume and locations as these studied populations), as recommended by guidelines, is associated with low risk for complications with potential significant benefit, especially in patients with risk factors for VTE and with stable bleed on repeat CT. Of note, data are scant on PTP agent of choice after ICH, with most studies including LMWH only, or both LMWH and UFH, as PTP options.

### 4.3. Non-Traumatic Subarachnoid Hemorrhage

SAH comprises 2–5% of all stroke types, with global incidence decreasing from 10.2 (1980) to 6.1 (2010) per 100,000 people [69], with a 90-day-case fatality rate of 30% [70,71].

The frequency of VTE in this population has been reported at 4–21% and is mostly diagnosed 1–3 weeks into hospitalization [72]. Patient-related risk factors include older age, male sex, black race, preexisting neurologic disorders, obesity, heart failure, coagulopathy, and disease-related risk factors (i.e., presence of paralysis, longer length of stay, mechanical ventilation after aneurysm treatment, and need for external ventricular drain [EVD] [72,73]).

#### 4.3.1. Timing of PTP and Outcomes

The results of five studies [74,75,76,77,78] suggested benefit with earlier initiation, with an associated decrease in VTE risk [75,76,77] or less unfavorable 12-month functional outcomes [75,76], with none definitively revealing an increased risk of bleeding complications. Two studies incorporated early repeat CT to inform the commencement of PTP [74,76]. Two RCTs published in the early 2000s suggested conflicting results, one finding that ENX < 24 h after aneurysm securement was associated with numerically more bleeding complications (four total events) vs. none with PCB [74], and the other finding ENX within 72 h to be associated with less delayed ischemic deficits (8.8% vs. 66.7%, *p* < 0.001), infarcts (3.5% vs. 28.3%, *p* < 0.001), and unfavorable functional outcomes (1y Glasgow Outcome Scale [GOS] 4.39 vs. 4.02, *p* = 0.017) [75]. Two single-center, retrospective studies concluded that delayed PTP initiation was significantly associated with worse outcomes, including increased risk of VTE and unfavorable GOS at discharge and 12 months without increased risk of bleeding complications (OR 4.8) [76,77].

#### 4.3.2. Choice of Agent for PTP and Outcomes

Only one retrieved study analyzed PTP choice (ENX vs. UFH < 24 h after coiling or <48 h after clipping), without finding an effect of agent on VTE or secondary ICH risk, and notably reported a 90-day mortality of 41% [79]. While data on the choice of agent for PTP after SAH are overall not definitive, there are data on the neuroprotective and anti-inflammatory properties of UFH, which, in addition to more facile reversibility, may tilt the favor toward the use of UFH in practice [79].

#### 4.3.3. Interpretation and Considerations

Existing data carry heterogeneity in the definition of early PTP, the agents studied and their dosing, and the timing of aneurysm securement in cases of aneurysmal SAH. There was also inconsistency in reporting important baseline characteristics (e.g., SAH severity scores, GCS) and in the utilization of varying endpoints. Further limiting generalizability was population heterogeneity, with large differences in reported mortality as well as significant practice change over time (particularly since the publication of the two retrieved RCTs [74,75]). Moreover, one RCT was likely underpowered to find more nuanced treatment effects, and the other was not powered for any single endpoint [75]. Other studies were limited by their retrospective, single-center design [77,79]. Still, the data point to the safety of practice with PTP < 24–72 h after SAH (and securing procedure), especially if informed by early stable CT findings, as outlined in guideline recommendations [6,11].

### 4.4. Spinal Cord Injury or Spinal Surgery

The incidence of traumatic SCI ranges from 12 to 58/million, with higher frequency in high-income countries [80]. The incidence of VTE in this patient population is high, with an estimated overall rate reported to exceed 18%, and still reaching 9% with the use of PTP regimens [81].

Risk factors for VTE following a traumatic SCI include older age (>45 years), male sex, smoking, prior VTE, concomitant lower limb or pelvic fractures, paraplegia or quadriplegia, diabetes mellitus, and failure to initiate mechanical compression prophylaxis [82]. While missed doses and a delay of PTP > 48 h in this patient population is also recognized to increase the risk of VTE, this may depend on individual risk factors rather than posing an independent risk [81].

#### 4.4.1. Timing of PTP and Outcomes

Twelve studies [81,83,84,85,86,87,88,89,90,91,92,93] found an association of early PTP initiation with decreased risk of VTE (largely < 24–48 h after injury or surgical intervention), without increased risk of bleeding complication, including two prospective observational studies [81,86] and one RCT [90]. One study indicated an increased risk of hematoma requiring return to the operating room (RTOR). This single-center cohort study of 8704 patients receiving elective spinal surgery without SCI (71% with spinal fusion procedure) analyzed UFH immediately vs. <24–48 h post-operatively. The adjusted results found no independent association of immediate PTP with lower VTE risk (OR 1.18, *p* = 0.44) but did find an increased risk of RTOR for hematoma (OR 3.1, *p* = 0.001) [92].

#### 4.4.2. Choice of Agent for PTP and Outcomes

Six analyses suggested a benefit with LMWH utilization vs. UFH [81,94,95,96,97,98]. In the secondary analysis of the prospective CLOTT study, which focused on an adjusted analysis of the effect of early PTP in SCI, an unadjusted analysis also found ENX to be associated with a decreased risk of VTE (7.5% vs. 21%, *p* = 0.003) [94]. Two other studies were large, multicenter, retrospective analyses with elective spinal surgeries included in one [95] and spinal trauma in another [98], with adjusted results suggesting independent association with LMWH benefits, including lower risks of blood transfusion, VTE, and mortality.

#### 4.4.3. Interpretation and Considerations

The interpretation of these data is challenged by the heterogeneity of the study populations, especially since the data include studies of elective surgery and urgent or emergent procedures, including spine trauma. Heterogeneity was also evident in the definition of early PTP, the reporting of bleeding complications (e.g., bleeding related to SCI reported in six studies with varying definitions, including intraspinal bleeding progression and need for surgical intervention) and the level of reporting, and the wide range of injury severity (e.g., only five studies characterized completed injuries and/or baseline injury severity score [ISS]). The relative paucity of bleeding complication events also raises the question of inadequate power to find differences and thus limits the detection of signals for safety concerns. Two randomized studies were considered [90,97] but three prospective studies were limited by their observational design [81,84,86], lacking multivariate regression analyses to address confounders in two cases. The rest were limited by their retrospective, and in some cases, single-center design. Still, these data suggest the safety and potential benefit of PTP initiation largely <24–72 h after injury/intervention (within 48 h in most studies) in patients without evidence of active bleeding, aligning with guideline recommendations [6,10,12].

Regarding PTP agent, the suggested benefit of ENX in one prospective study was a result unadjusted for confounding variables, and timing of PTP was not reported in detail [94]. Still, large multicenter retrospective studies corroborated benefit, though still limited by their retrospective design. The limited nature of the data allows providers their choice of PTP, though LMWH could be considered in select patients based on the weakly supported signal for benefit without evident harm.

### 4.5. Neurosurgical Intervention

The observed frequency of VTE in patients undergoing neurosurgical intervention is reported at 3–26%, with the highest overall risk in patients with high-grade glioma [99].

Risk factors for VTE in this subtype can be classified into patient related (i.e., older age, obesity, lower limb paresis, and history of VTE); tumor disease type, including glioblastoma, IDH1, intratumor thrombus); and treatment related (namely biopsy, tumor resection, and use of corticosteroids/anti VEGF therapy). Certain biomarkers including leukocytosis, thrombocytopenia, elevated factor VIII activity, and increased D-dimer levels have also been identified as related risk factors [100].

#### 4.5.1. Timing of PTP and Outcomes

Early PTP was associated with reduced incidence of VTE in 10 studies on the topic and reduced mortality risk in another (<24–72 h postoperatively) [101,102,103,104,105,106,107,108,109,110], with two studies observing an increased risk of bleeding complication [102,105]. One multicenter, retrospective study analyzed 4951 patients with blunt, isolated TBI (91% with ICH) requiring neurosurgical intervention (78% with craniotomy/craniectomy) <24 h after admission with a median time to PTP of 3 d. Each day of PTP delay was independently associated with increased VTE risk (OR 1.08 per day, *p* < 0.05), but each day of delay from day 1–3 was associated with a 28% decreased risk of repeated neurosurgery (OR 0.72 per day, *p* < 0.05). In patients who underwent intracranial monitor/drain insertions, each day of delay was associated with a decreased risk of death (OR 0.94 per day, *p* < 0.05) [102]. A single-center, retrospective study of 614 patients undergoing elective craniotomy for tumor resection who received PTP < 24 vs. <48 h post-operatively found earlier PTP to be associated with increased risk of hemorrhagic complication only in the subgroup aged >70 years old and with glioblastomas and subtotal resection (OR 12.98, *p* < 0.05) [105].

#### 4.5.2. Choice of Agent for PTP and Outcomes

None of the four studies retrieved found a benefit from one PTP choice over another [107,111,112,113]. A single-center, retrospective, propensity score matched study of 2901 elective intracranial surgery patients found no difference in VTE but did find an increased prevalence of clinically significant ICH with ENX compared to UFH (3.4% vs. 0.5%, *p* = 0.008) [112].

#### 4.5.3. Interpretation and Considerations

The analysis of these studies is complicated by the surgical heterogeneity of the included study populations, including craniotomy and craniectomy, drain and intracranial monitor placement, and surgeries with elective and urgent timing. Definitions for early PTP as well as bleeding complications are also heterogenous (sometimes vaguely defined vs. ICH progression or need for neurosurgical intervention). The characteristics of the studied populations were reported inconsistently, restricting the assessment of the severity of presenting neurosurgical injury, further limiting generalizability. The data, though scant, suggest that PTP < 24–72 h post-operatively could be considered (including in appropriate patients <24 h after elective craniotomy) [6]. The signal for a potentially increased risk for repeat NI and mortality, especially within the first 72 h, should highlight the careful patient-specific risk vs. benefit estimation providers should practice when considering PTP in these patients. PTP choice was analyzed sparsely, without finding an effect of agent on VTE risk [111], with one study suggesting the potential for ICH risk with ENX, demonstrating the need for individualized decision making.

### 4.6. PTP Interruption and VTE Risk

While our main questions centered around initiation time and agent for PTP, an important clinical consideration is the consistency of PTP and the effect of missed PTP doses on VTE risk. Five retrospective studies were retrieved with such analyses, all suggesting an increased VTE risk (three in TBI, one in SCI, and one in neurosurgery with SAH) [21,27,81,114,115]. Multivariate analysis in two retrospective TBI studies suggested an association of missed doses and increased VTE risk (OR 1.08–7.07) [27,115], and univariate analysis in another suggested 4–6 missed doses was associated with the highest risk of VTE (OR 4.1, *p* = 0.005) [21]. A secondary analysis of SCI patients in the CLOTT study reported VTE in 14.7% with interrupted and 7.5% with continuous PTP (*p* = 0.038), though multivariate analysis did not find an independent association (OR 1.34, *p* = 0.48) [81]. A single-center, propensity score-matched analysis of SAH patients with EVD found that holding >1 PTP dose for EVD removal was associated with an increased VTE risk (OR 4.8, *p* = 0.009) [114]. The decision to hold PTP is a common clinical scenario with strikingly sparse data to inform practice, and further study and focus on this topic would aid in comprehensive decision making surrounding appropriate PTP utilization.

## 5. Limitations of Existing Data

The majority of available studies are retrospective and carry the inherent limitations of such analyses. The fewer prospective studies available are limited by smaller sample sizes. Underreporting of baseline characteristics, disease severity, methods on assessment of efficacy/safety outcomes (VTE or bleeding), or dosing often result in an inability to conduct more robust analyses (i.e., multivariate logistic regression). The heterogeneity of populations and intervention/outcome definitions complicates generalization and adaptation into clinical practice. Further, most studies appeared underpowered to analyze safety outcomes.

## 6. Conclusions

In summary, guidance for the exact time of initiation and specific choice of PTP after neurocritical illness is limited. Individual disease-specific risk factors, particularly the presence of immobility or paralysis, and higher injury severity, should prompt an urgent consideration of PTP, as these increase the risk of VTE. The available practice data also indicate that the feared risk of HE may lead to delays in starting PTP, especially in patients with a higher risk of VTE, while most data indicate that PTP within 24–48 h does not increase hemorrhagic risk. The currently available data are limited in interpretation due to methodology, heterogeneity, and failure to account for the relevant high risk of early death due to withdrawal of life-sustaining therapies in the neurocritically ill.

## Figures and Tables

**Table 1:** Summary of most recent consensus guideline recommendations for prophylaxis of venous thromboembolism in neurocritically ill patients. This table provides a comprehensive, disease-based overview of existing guidelines or consensus recommendations for venous thromboembolism in neurocritically ill patients. Of note, all recommendations from these guidelines are based on a reported low-to-medium quality of evidence. * *Prophylaxis of Venous Thrombosis in Neurocritical Care Patients: An Evidence-Based Guideline: A Statement for Healthcare Professionals from the Neurocritical Care Society*; ** *Prophylaxis of Venous Thrombosis in Neurocritical Care Patients: An Executive Summary of Evidence-Based Guidelines: A Statement for Healthcare Professionals From the Neurocritical Care Society and Society of Critical Care Medicine*; ^†^ *2023 Guideline for the Management of Patients With Aneurysmal Subarachnoid Hemorrhage: A Guideline From the American Heart Association/American Stroke Association*; ^‡^ *2022 Guideline for the Management of Patients With Spontaneous Intracerebral Hemorrhage: A Guideline From the American Heart Association/American Stroke Association*; ^¥^ *Prevention of Venous Thromboembolism in Individuals with Spinal Cord Injury: Clinical Practice Guidelines for Health Care Providers, 3rd ed*; ^††^ *Updated guidelines to reduce venous thromboembolism in trauma patients: A Western Trauma Association critical decisions algorithm*; ^‡‡^ *Guidelines for the Management of Severe Traumatic Brain Injury, Fourth Edition*; ^¥¥^ *Best practices guidelines: the management of traumatic brain injury; aSAH = aneurysmal subarachnoid hemorrhage*; CrCL = creatinine clearance; ICH = intracerebral hemorrhage; LMWH = low-molecular weight heparin; SCI = spinal cord injury; UFH = unfractionated heparin; VTE = venous thromboembolism.

Guideline (Publication Year)	Recommendations	
	Timing	Agent
**aSAH**		
NCS 2016 *	≥24 h after aneurysm securement by open surgical approach or by endovascular coiling	UFH recommended for PTP
NCS/SCCM 2017 **	≥24 h after aneurysm securement by open surgical approach or by endovascular coiling	UFH recommended for PTP
**AHA/ASA 2023 ^†^**	After aneurysm securement by open surgical approach or by endovascular coiling	No recommendation
**ICH**		
**NCS 2016** *	Within 48 h of hospital admission with stable hematoma and no ongoing coagulopathy	LMWH or UFH
** NCS/SCCM ** ** 2017 ** **	Within 48 h of hospital admission with stable hematoma and no ongoing coagulopathy	LMWH or UFH
** AHA/ASA 2022 ^‡^**	At 24–48 h from ICH onset	LMWH or UFH
**Neurosurgical Intervention **		
**NCS 2016** *	**Standard Elective Spine Surgery**	
	No recommendation	LMWH (combined with mechanical specifically with increased risk of VTE), with UFH only as an alternative to other methods because of increased risk of bleeding
	**Complicated Spinal Surgery**	
	No recommendation	LMWH or UFH
	**Elective Craniotomy (with or without glioma resection)**	
	Within 24 h after craniotomy	LMWH or UFH
	**Elective Intracranial/Intra-arterial Procedures**	
	Immediate	LMWH or UFH
** NCS/SCCM ** ** 2017 ** **	**Standard Elective Spine Surgery**	
	No recommendation	LMWH (combined with mechanical specifically with increased risk of VTE), with UFH only as an alternative to other methods because of increased risk of bleeding
	**Complicated Spinal Surgery**	
	No recommendation	LMWH or UFH
	**Elective Craniotomy (with or without glioma resection)**	
	Within 24 h after craniotomy	LMWH or UFH
	**Elective Intracranial/Intra-arterial Procedures**	
	Immediate	LMWH or UFH
**SCI**		
** NCS ** 2016 *	Early as possible, within 72 h of injury; as soon as bleeding is controlled	LMWH or adjusted dose UFH
**Consortium for Spinal Cord Medicine 2016** **^ ¥ ^**	After there is no evidence of active bleeding	LMWH recommended for PTP; UFH recommended against as low-dose or adjusted dose
NCS/SCCM 2017 **	Early as possible, within 72 h of injury; as soon as bleeding is controlled	LMWH or adjusted dose UFH
**WTA 2020** **^ †† ^**	Within 24 h with moderate–high-risk VTE and stabilization of spinal cord injury	LMWH 30 mg q12 h (with CrCL ≥ 30 mL/min), preferable to UFH
**TBI**		
** NCS ** 2016 *	Within 24 h of TBI or within 24 h after craniotomy; within 24–48 h in patients with TBI and ICH or 24 h after craniotomy	LMWH or UFH,
NCS/SCCM 2017 **	Within 24 h of TBI or within 24 h after craniotomy; within 24–48 h in patients with TBI and ICH or 24 h after craniotomy	LMWH or UFH,
**Brain Trauma Foundation 2017** ^‡‡^	No recommendation	LMWH or low-dose UFH; however, there is noted to be an increased risk of expansion of ICH
**WTA 2020** **^ †† ^**	Within 24 h with moderate–high-risk VTE and no TBI progression on follow-up CT	LMWH, ENX 30 mg q12 h (with CrCL ≥ 30 mL/min), preferable to UFH
**American College of Surgeons 2024** **^ ¥¥ ^**	Within 24 h with low-risk nonoperative TBI and no TBI progression on follow-up CT; within 24–48 h with moderate–severe risk nonoperative TBI and no TBI progression on follow-up CT; within 24–48 h after craniotomy/craniectomy for TBI and no ICH progression on postoperative CT	LMWH preferred over UFH

**Table 2:** Comparison of unfractionated heparin and enoxaparin. AUC = area under curve; CrCl = creatinine clearance; HIT = heparin induced thrombocytopenia; kg = kilogram; mg = milligram; MW = molecular weight; RES = reticuloendothelial system; UFH = unfractionated heparin.

	UFH	Enoxaparin
**Bioavailability**	Variable	100%
**Mean MW (range) (kDa)**	16 (4–30)	4.5 (mostly 2–8)
**Proportion with both anti-Xa and anti-IIa activity**	95%	<30%
**Metabolism**	RES primarily in liver and spleen	Hepatic (desulfation and/or depolymerization to lower weight molecules with very low potency)
**Excretion**	Urine (small amounts as unchanged drug); elimination of therapeutic doses occurs rapidly via nonrenal mechanisms	Urine (clearance decreased by 30% and AUC increased 65% with CrCL < 30 mL/min)
**Pharmacokinetics**	First-order (with time and dose dependence of anti-Xa/anti-IIa effects)	Mixed-order kinetic behavior
**Half-life elimination, plasma**	1–2 h	4.5–7 h (based on anti-Xa activity) (duration 40 mg dose ~ 12 h based on anti-Xa activity)
**Usual subcutaneous prophylaxis dosing interval**	5000 units q8h-q12h	30–40 mg q12h-q24h
**Maximum neutralization by protamine (%)**	100	60–75
**Risk of HIT**	2.60%	0.20%

## Data Availability

All data used in this article were directly obtained from public databases like PubMed and EMBASE.

## Supplementary Materials

The following supporting information can be downloaded at https://www.mdpi.com/article/10.3390/jcm14134434/s1, Literature review table.

## Abbreviations

The following abbreviations are used in this manuscript:

AIS	Abbreviated injury scale
CT	Computer tomography
DVT	Deep vein thrombosis
ENX	Enoxaparin
EVD	External ventricular drain
GCS	Glasgow coma scale
GOS	Glasgow outcome scale
HE	Hematoma expansion
ICH	Intracranial hemorrhage
ICP	Intracranial pressure
IDH1	Isocitrate dehydrogenase
IVC	Infravena cava
NCS	Neurocritical care society
NI	Neurosurgical intervention
OR	Odds Ratio
PCB	Placebo
PE	Pulmonary embolism
PTP	Pharmacologic thromboprophylaxis
RCT	Randomized control trial
RTOR	Return to OR
SAH	Subarachnoid hemorrhage
SCI	Spinal cord injury
TBI	Traumatic brain injury
UFH	Unfractionated heparin
VEGF	Vascular endothelial growth factor
VTE	Venous thromboembolism
