# Supplementary material for: Venous Thromboembolism Prophylaxis in the Neurocritically Ill Population
Source: J Clin Med. 2025 Jun 22;14(13):4434. doi: 10.3390/jcm14134434 (PMC12249765; doi:10.3390/jcm14134434)
Supplement: Supplementary file 1 [file jcm-14-04434-s001.zip › jcm-3672439-supplementary.pdf]

Search No.	PubMed Search Query
26	#23 NOT #24 AND English Filter Applied
25	#23 NOT #24
24	"Rats"[Mesh] OR "Mice"[Mesh] OR "mus genus"[tiab] OR "mice"[tiab] OR "mouse"[tiab] OR "newborn mice"[tiab] OR "rattus"[tiab] OR "rat"[tiab] OR "rats"[tiab] OR animal*[tiab]
23	#21 NOT #22
22	("Animals"[Mesh] NOT ("Animals"[Mesh] AND "Humans"[Mesh]))
21	#3 AND #4 AND #20
20	#5 OR #6 OR #7 OR #8 OR #9 OR #10 OR #11 OR #12 OR #13 OR #14 OR #15 OR #16 OR #17 OR #18 OR #19
19	"Neurosurgery"[Mesh] OR "neurologic surgery"[tiab] OR "neurological surgery"[tiab] OR "neurosurgery"[tiab] OR "neurosurgical emergency"[tiab] OR "neurosurgical operation"[tiab] OR "neurosurgical patient"[tiab] OR "neurosurgical procedure"[tiab] OR "neurosurgical procedures"[tiab]
18	"Spinal Cord Injuries"[Mesh] OR "injury, spinal cord"[tiab] OR "spinal cord injuries"[tiab] OR "spinal cord injury"[tiab] OR "spinal cord trauma"[tiab] OR "trauma, spinal cord"[tiab]
17	"Cerebral Hemorrhage, Traumatic"[Mesh] OR "intraparenchymal hemorrhage"[tiab]
16	"Hematoma, Epidural, Cranial"[Mesh] OR "cranial epidural haematoma"[tiab] OR "cranial epidural hematoma"[tiab] OR "epidural haematoma"[tiab] OR "epidural hematoma"[tiab] OR "extradural haematoma"[tiab] OR "extradural hematoma"[tiab] OR "haematoma, epidural"[tiab] OR "haematoma, epidural, cranial"[tiab] OR "haematoma, extradural"[tiab] OR "hematoma, epidural"[tiab] OR "hematoma, epidural, cranial"[tiab] OR "hematoma, extradural"[tiab]
15	"Hematoma, Subdural"[Mesh] OR "Hematoma, Subdural, Intracranial"[Mesh] OR "Hematoma, Subdural, Acute"[Mesh] OR "Hematoma, Subdural, Chronic"[Mesh] OR "Hematoma, Subdural, Spinal"[Mesh] OR "acute subdural haematoma"[tiab] OR "acute subdural hematoma"[tiab] OR "chronic subdural haematoma"[tiab] OR "chronic subdural haematomata"[tiab] OR "chronic subdural hematoma"[tiab] OR "chronic subdural hematomata"[tiab] OR "haematoma, subdural"[tiab] OR "haematoma, subdural, acute"[tiab] OR "haematoma, subdural, chronic"[tiab] OR "haematoma, subdural, intracranial"[tiab] OR "haemorrhage, subdural"[tiab] OR "haemorrhagic pachymeningitis"[tiab] OR "hematoma, subdural"[tiab] OR "hematoma, subdural, acute"[tiab] OR "hematoma, subdural, chronic"[tiab] OR "hematoma, subdural, intracranial"[tiab] OR "hemorrhage, subdural"[tiab] OR "hemorrhagic pachymeningitis"[tiab] OR "intracranial subdural haematoma"[tiab] OR "intracranial subdural haematomas"[tiab] OR "intracranial subdural haematomata"[tiab] OR "intracranial subdural hematoma"[tiab] OR "intracranial subdural hematomas"[tiab] OR "intracranial subdural hematomata"[tiab] OR "pachymeningiosis haemorrhagica interna"[tiab] OR "pachymeningitis haemorrhagica"[tiab] OR "subdural bleeding"[tiab] OR "subdural haematoma"[tiab] OR "subdural haemorrhage"[tiab] OR "subdural hematoma"[tiab] OR "subdural hemorrhage"[tiab] OR "subepidural haematoma"[tiab] OR "subepidural hematoma"[tiab]
14	"experimental sah subarachnoid hemorrhage"[tiab] OR "experimental subarachnoid bleeding"[tiab] OR "experimental subarachnoid haemorrhage"[tiab] OR "experimental subarachnoid hemorrhage"[tiab] OR "experimentally induced sah subarachnoid hemorrhage"[tiab] OR "experimentally induced subarachnoid bleeding"[tiab] OR "experimentally induced subarachnoid haemorrhage"[tiab] OR "experimentally induced subarachnoid hemorrhage"[tiab]
13	"Subarachnoid Hemorrhage"[Mesh] OR "Subarachnoid Hemorrhage, Traumatic"[Mesh] OR "aneurysmal subarachnoid haemorrhage"[tiab] OR "aneurysmal subarachnoid hemorrhage"[tiab] OR "arachnoid haemorrhage, brain"[tiab] OR "arachnoid hemorrhage, brain"[tiab] OR "arachnoidal bleeding"[tiab] OR "arachnoidal haemorrhage"[tiab] OR "arachnoidal haemorrhage, brain"[tiab] OR "arachnoidal hemorrhage"[tiab] OR "arachnoidal hemorrhage, brain"[tiab] OR "bleeding, subarachnoid"[tiab] OR "brain arachnoid haemorrhage"[tiab] OR "brain arachnoid hemorrhage"[tiab] OR "haemorrhage, subarachnoid"[tiab] OR "hemorrhage, subarachnoid"[tiab] OR "spontaneous subarachnoid haemorrhage"[tiab] OR "spontaneous subarachnoid hemorrhage"[tiab] OR "subarachnoid bleeding"[tiab] OR "subarachnoid blood"[tiab] OR "subarachnoid haematoma"[tiab] OR "subarachnoid haemorrhage"[tiab] OR "subarachnoid haemorrhage, brain"[tiab] OR "subarachnoid haemorrhage, traumatic"[tiab] OR "subarachnoid hematoma"[tiab] OR "subarachnoid hemorrhage"[tiab] OR

	"subarachnoid hemorrhage, brain"[tiab] OR "subarachnoid hemorrhage, traumatic"[tiab] OR "subarachnoid hemorrhagia"[tiab] OR "subarachnoidal bleeding"[tiab] OR "subarachnoidal haemorrhage"[tiab] OR "subarachnoidal hemorrhage"[tiab] OR "traumatic subarachnoid haemorrhage"[tiab] OR "traumatic subarachnoid hemorrhage"[tiab]
12	"massive cerebral bleeding"[tiab] OR "massive cerebral haemorrhage"[tiab] OR "massive cerebral hemorrhage"[tiab] OR "massive intracerebral bleeding"[tiab] OR "massive intracerebral haemorrhage"[tiab] OR "massive intracerebral hemorrhage"[tiab]
11	"cerebellar haemorrhage"[tiab] OR "cerebellar hemorrhage"[tiab] OR "cerebellum haemorrhage"[tiab] OR "cerebellum hemorrhage"[tiab] OR "haemorrhage, cerebellum"[tiab] OR "hemorrhage, cerebellum"[tiab]
10	"brain ventricle bleeding"[tiab] OR "brain ventricle haemorrhage"[tiab] OR "brain ventricle hemorrhage"[tiab] OR "cerebral intraventricular hemorrhage"[tiab]
9	"Intracranial Hemorrhages"[Mesh] OR "Brain Hemorrhage, Traumatic"[Mesh] OR "Cerebral Hemorrhage"[Mesh] OR "bleeding, corpus callosum"[tiab] OR "brain bleeding"[tiab] OR "brain haemorrhage"[tiab] OR "brain haemorrhage, traumatic"[tiab] OR "brain hemorrhage"[tiab] OR "brain hemorrhage, traumatic"[tiab] OR "brain microhaemorrhage"[tiab] OR "brain microhemorrhage"[tiab] OR "brain stem haemorrhage, traumatic"[tiab] OR "brain stem hemorrhage, traumatic"[tiab] OR "cerebral haemorrhage"[tiab] OR "cerebral haemorrhage, traumatic"[tiab] OR "cerebral hemorrhage"[tiab] OR "cerebral hemorrhage, traumatic"[tiab] OR "cerebral microbleed"[tiab] OR "corpus callosum bleeding"[tiab] OR "corpus callosum haemorrhage"[tiab] OR "corpus callosum hemorrhage"[tiab] OR "encephalorrhagia"[tiab] OR "haemorrhage, brain"[tiab] OR "haemorrhage, intracranial"[tiab] OR "haemorrhagic apoplexy"[tiab] OR "haemorrhagic stroke"[tiab] OR "haemorrhagic stroke intracerebral bleeding"[tiab] OR "hematencephalon"[tiab] OR "hemorrhage, brain"[tiab] OR "hemorrhage, intracranial"[tiab] OR "hemorrhagic apoplexy"[tiab] OR "hemorrhagic stroke"[tiab] OR "hemorrhagic stroke intracerebral bleeding"[tiab] OR "hypertensive intracranial haemorrhage"[tiab] OR "hypertensive intracranial hemorrhage"[tiab] OR "intracerebral bleeding"[tiab] OR "intracerebral haemorrhage"[tiab] OR "intracerebral hemorrhage"[tiab] OR "intracortical haemorrhage"[tiab] OR "intracortical hemorrhage"[tiab] OR "intracranial bleeding"[tiab] OR "intracranial haemorrhage"[tiab] OR "intracranial haemorrhage, hypertensive"[tiab] OR "intracranial haemorrhage, traumatic"[tiab] OR "intracranial haemorrhages"[tiab] OR "intracranial hemorrhage"[tiab] OR "intracranial hemorrhage, hypertensive"[tiab] OR "intracranial hemorrhage, traumatic"[tiab] OR "intracranial hemorrhages"[tiab] OR "intraventricular haemorrhage"[tiab] OR "intraventricular hemorrhage"[tiab] OR "periventricular haemorrhage"[tiab] OR "periventricular hemorrhage"[tiab] OR "posterior fossa haemorrhage"[tiab] OR "posterior fossa hemorrhage"[tiab] OR "traumatic brain haemorrhage"[tiab] OR "traumatic brain hemorrhage"[tiab] OR "traumatic brain stem haemorrhage"[tiab] OR "traumatic brain stem hemorrhage"[tiab] OR "traumatic cerebral haemorrhage"[tiab] OR "traumatic cerebral hemorrhage"[tiab] OR "traumatic intracranial haemorrhage"[tiab] OR "traumatic intracranial hemorrhage"[tiab]
8	"nicu neurologic"[tiab] OR "nicus neurologic"[tiab] OR "neuro-icu"[tiab] OR "neuro-icus"[tiab] OR "neuro-critical care unit"[tiab] OR "neuro-intensive care unit"[tiab] OR "neuroicu"[tiab] OR "neuroicus"[tiab] OR "neurocritical care unit"[tiab] OR "neurointensive care unit"[tiab] OR "neurologic intensive care unit"[tiab] OR "neurological intensive care unit"[tiab] OR "neuroscience icu"[tiab] OR "neuroscience critical care unit"[tiab] OR "neurosciences critical care unit"[tiab]
7	"child traumatic brain injury"[tiab] OR "childhood traumatic brain injury"[tiab] OR "paediatric tbi"[tiab] OR "paediatric traumatic brain injury"[tiab] OR "pediatric tbi"[tiab] OR "pediatric traumatic brain injury"[tiab]
6	"Chronic Traumatic Encephalopathy"[Mesh] OR "chronic traumatic brain injury"[tiab] OR "chronic traumatic encephalopathy"[tiab] OR "dementia pugulistica"[tiab]
5	"Brain Injuries, Traumatic"[Mesh] OR "Brain Concussion"[Mesh] OR "brain injuries, traumatic"[tiab] OR "brain lesion, traumatic"[tiab] OR "brain system trauma"[tiab] OR "brain trauma"[tiab] OR "cerebral trauma"[tiab] OR "cerebrovascular trauma"[tiab] OR "encephalopathy, traumatic"[tiab] OR "mild traumatic brain injury"[tiab] OR "organic cerebral trauma"[tiab] OR "posttraumatic encephalopathy"[tiab]

	OR "traumatic brain injuries"[tiab] OR "traumatic brain injury"[tiab] OR "traumatic brain lesion"[tiab] OR "traumatic cerebral lesion"[tiab] OR "traumatic encephalopathy"[tiab]
4	"Enoxaparin"[Mesh] OR "enoxaparin sodium" [Supplementary Concept] OR "arovi"[tiab] OR "clexan"[tiab] OR "clexane"[tiab] OR "clexane 40"[tiab] OR "clexane forte"[tiab] OR "clexane multidose"[tiab] OR "clexane t"[tiab] OR "colevance"[tiab] OR "crusia"[tiab] OR "decipar"[tiab] OR "enoxaparin"[tiab] OR "enoxaparin sodium"[tiab] OR "ghemaxan"[tiab] OR "hepaxane"[tiab] OR "inhixa"[tiab] OR "klexane"[tiab] OR "ledraxen"[tiab] OR "losima"[tiab] OR "lovenox"[tiab] OR "lovenox preservative free"[tiab] OR "neoparin"[tiab] OR "neoparin-nx"[tiab] OR "percolozin"[tiab] OR "pk 10169"[tiab] OR "pk10169"[tiab] OR "qualiop klinik"[tiab] OR "rovinadil"[tiab] OR "rp 54563"[tiab] OR "rp54563"[tiab] OR "thorinane"[tiab] OR "Heparin"[Mesh] OR "alpha heparin"[tiab] OR "ammonium heparinate"[tiab] OR "benzalkonium heparin"[tiab] OR "beparine"[tiab] OR "clarin"[tiab] OR "contusol"[tiab] OR "disebrin"[tiab] OR "eleparon"[tiab] OR "elheparin"[tiab] OR "elheparon"[tiab] OR "endogenous heparin"[tiab] OR "epiheparin"[tiab] OR "gag 98"[tiab] OR "helberina"[tiab] OR "hep flush kit"[tiab] OR "hep lock"[tiab] OR "hep-lock"[tiab] OR "hep-pak cvc"[tiab] OR "hepaflex"[tiab] OR "hepalean"[tiab] OR "heparin"[tiab] OR "heparin injection b.p."[tiab] OR "heparin leo"[tiab] OR "heparin lock flush"[tiab] OR "heparin lock flush plus sodium chloride"[tiab] OR "heparin lock flush preservative free"[tiab] OR "heparin monosulfate"[tiab] OR "heparin monosulphate"[tiab] OR "heparin novo"[tiab] OR "heparin ointment"[tiab] OR "heparin potassium"[tiab] OR "heparin sodium"[tiab] OR "heparin sodium 1, 000 units and sodium chloride 0.9%"[tiab] OR "heparin sodium 1, 000 units in dextrose 5%"[tiab] OR "heparin sodium 1, 000 units in sodium chloride 0.9%"[tiab] OR "heparin sodium 10, 000 units in dextrose 5%"[tiab] OR "heparin sodium 10, 000 units in sodium chloride 0.45%"[tiab] OR "heparin sodium 10, 000 units in sodium chloride 0.9%"[tiab] OR "heparin sodium 12, 500 units in dextrose 5%"[tiab] OR "heparin sodium 12, 500 units in sodium chloride 0.45%"[tiab] OR "heparin sodium 12, 500 units in sodium chloride 0.9%"[tiab] OR "heparin sodium 2, 000 units and sodium chloride 0.9%"[tiab] OR "heparin sodium 2, 000 units in dextrose 5%"[tiab] OR "heparin sodium 2, 000 units in sodium chloride 0.9%"[tiab] OR "heparin sodium 20, 000 units and dextrose 5%"[tiab] OR "heparin sodium 20, 000 units in dextrose 5%"[tiab] OR "heparin sodium 25, 000 units and dextrose 5%"[tiab] OR "heparin sodium 25, 000 units in dextrose 5%"[tiab] OR "heparin sodium 25, 000 units in sodium chloride 0.45%"[tiab] OR "heparin sodium 25, 000 units in sodium chloride 0.9%"[tiab] OR "heparin sodium 5, 000 units and sodium chloride 0.9%"[tiab] OR "heparin sodium 5, 000 units in dextrose 5%"[tiab] OR "heparin sodium 5, 000 units in sodium chloride 0.45%"[tiab] OR "heparin sodium 5, 000 units in sodium chloride 0.9%"[tiab] OR "heparin sodium b braun"[tiab] OR "heparin sodium preservative free"[tiab] OR "heparin subcutaneous"[tiab] OR "heparin sulfate"[tiab] OR "heparin sulfuric acid"[tiab] OR "heparin sulphate"[tiab] OR "heparina"[tiab] OR "heparina leo"[tiab] OR "heparinate sodium"[tiab] OR "heparine"[tiab] OR "heparine choay"[tiab] OR "heparine novo"[tiab] OR "heparinic acid"[tiab] OR "heparitin monosulfate"[tiab] OR "heparitin monosulphate"[tiab] OR "hepcon"[tiab] OR "hepflush-10"[tiab] OR "hepsal"[tiab] OR "inhepar"[tiab] OR "inviclot"[tiab] OR "lipo hepin"[tiab] OR "lipo-hepin"[tiab] OR "lipohepin"[tiab] OR "liquaemin"[tiab] OR "liquaemin lock flush"[tiab] OR "liquaemin sodium"[tiab] OR "liquaemin sodium preservative free"[tiab] OR "liquemin"[tiab] OR "liquemine"[tiab] OR "menaven"[tiab] OR "monoparin"[tiab] OR "mucoitin polysulfate"[tiab] OR "mucoitin polysulfate ester"[tiab] OR "mucoitin polysulphate"[tiab] OR "mucoitin polysulphate ester"[tiab] OR "mucoitin sodium polysulfate"[tiab] OR "mucoitin sodium polysulphate"[tiab] OR "multiparin"[tiab] OR "nevparin"[tiab] OR "noparin"[tiab] OR "panheparin"[tiab] OR "panhepin"[tiab] OR "panheprin"[tiab] OR "parinix"[tiab] OR "phlebotroy qps"[tiab] OR "praecivenin"[tiab] OR "pularin"[tiab] OR "sodium heparin"[tiab] OR "thrombareduct"[tiab] OR "thrombo vetren"[tiab] OR "thromboliquin"[tiab] OR "thromboliquine"[tiab] OR "thrombophlogat"[tiab] OR "thrombophob"[tiab] OR "thrombophob gel"[tiab] OR "thromboreduct"[tiab] OR "thrombosamine"[tiab] OR "thrombosamine heparin"[tiab] OR "thrombosamine heparine"[tiab] OR "unfractionated heparin"[tiab] OR "uniparin"[tiab] OR "vetren"[tiab] OR "vister"[tiab] OR "vr 496"[tiab] OR "vr496"[tiab]
3	#1 OR #2

2	"Venous Thromboembolism"[Mesh] OR "thromboembolism, venous"[tiab] OR "vein thromboembolism"[tiab] OR "venous thromboembolism"[tiab] OR "Venous Thrombosis"[Mesh] OR "Upper Extremity Deep Vein Thrombosis"[Mesh] OR "dvt deep vein thrombosis"[tiab] OR "acute dvt"[tiab] OR "acute deep venous thrombosis"[tiab] OR "deep thrombo-phlebitis"[tiab] OR "deep thrombophlebitis"[tiab] OR "deep vein blood clots"[tiab] OR "deep vein thrombophlebitis"[tiab] OR "deep vein thrombosis"[tiab] OR "deep vein thrombus"[tiab] OR "deep venous thrombophlebitis"[tiab] OR "deep venous thrombosis"[tiab] OR "deep venous thrombus"[tiab] OR "recurrent dvt"[tiab] OR "thrombosis, acute deep venous"[tiab] OR "may thurner syndrome"[tiab] OR "may-thurner syndrome"[tiab] OR "lower extremity deep vein thrombosis"[tiab] OR "ovarian vein thromboses"[tiab] OR "ovarian vein thrombosis"[tiab] OR "ovarian venous thrombosis"[tiab] OR "ovary vein thrombosis"[tiab] OR "Thrombophlebitis"[Mesh] OR "phlegmasia alba"[tiab] OR "phlegmasia alba dolens"[tiab] OR "white leg thrombosis"[tiab] OR "white phlegmasia"[tiab] OR "gregoire`s blue leg"[tiab] OR "gregoire`s blue phlebitis"[tiab] OR "gregoire`s blue thrombophlebitis"[tiab] OR "blue phlebitis"[tiab] OR "blue phlegmasia"[tiab] OR "blue thrombophlebitis"[tiab] OR "phlegmasia caerulea"[tiab] OR "phlegmasia caerulea dolens"[tiab] OR "phlegmasia cerulea"[tiab] OR "phlegmasia cerulea dolens"[tiab] OR "phlegmasia coerulea"[tiab] OR "phlegmasia coerulea dolens"[tiab] OR "Upper Extremity Deep Vein Thrombosis"[Mesh] OR "paget schroetter disease"[tiab] OR "paget schroetter syndrome"[tiab] OR "paget schrotter syndrome"[tiab] OR "paget von schroetter disease"[tiab] OR "paget von schroetter syndrome"[tiab] OR "schroetter paget syndrome"[tiab] OR "axillary vein thrombosis"[tiab] OR "deep vein thrombosis of the upper extremity"[tiab] OR "effort thrombosis"[tiab] OR "subclavian vein thrombosis"[tiab] OR "subclavian venous thrombosis"[tiab] OR "thrombosis of the subclavian vein"[tiab] OR "upper extremity deep vein thrombosis"[tiab] OR "upper extremity deep venous thrombosis"[tiab] OR "upper extremity thrombosis"[tiab] OR "upper limb deep vein thrombosis"[tiab] OR "Pulmonary Embolism"[Mesh] OR "chronic lung embolism"[tiab] OR "embolism, lung"[tiab] OR "lung embolism"[tiab] OR "lung embolization"[tiab] OR "lung embolus"[tiab] OR "lung embolus recurrence"[tiab] OR "lung emboly"[tiab] OR "lung microembolism"[tiab] OR "lung microembolization"[tiab] OR "lung microembolus"[tiab] OR "lung thromboembolism"[tiab] OR "microembolus, lung"[tiab] OR "pulmonary embolism"[tiab] OR "pulmonary embolization"[tiab] OR "pulmonary embolus"[tiab] OR "pulmonary microembolism"[tiab] OR "pulmonary thromboembolic disease"[tiab] OR "pulmonary thromboembolism"[tiab] OR "thromboembolism, lung"[tiab]
1	"prophylaxis, thrombosis"[tiab] OR "thrombo-prophylaxis"[tiab] OR "thromboprophylaxis"[tiab] OR "thrombosis prevention"[tiab] OR "thrombosis prophylaxis"[tiab] OR "disease prophylaxis"[tiab] OR "prophylactic institution"[tiab] OR "prophylactic management"[tiab] OR "prophylactic medication"[tiab] OR "prophylactic therapy"[tiab] OR "prophylactic treatment"[tiab] OR "prophylaxis"[tiab] OR "Chemoprevention"[Mesh] OR "chemoprevention"[tiab] OR "chemoprophylaxis"[tiab] OR "chemoprophylaxis, tuberculosis"[tiab] OR "tuberculosis chemoprophylaxis"[tiab] OR "tuberculous chemoprophylaxis"[tiab]
